# Acceptability, internal consistency and test–retest reliability of scales to assess parental and nursery staff’s self-efficacy, motivation and knowledge in relation to pre-school children’s nutrition, oral health and physical activity

**DOI:** 10.1017/S1368980018004111

**Published:** 2019-02-14

**Authors:** Kaiseree Dias, James White, Chris Metcalfe, Ruth Kipping, Angeliki Papadaki, Russell Jago

**Affiliations:** 1 Population Health Sciences, Bristol Medical School, University of Bristol, Oakfield House, Oakfield Grove, Bristol BS8 2BN, UK; 2 Centre for Trials Research, School of Medicine, Cardiff University, Cardiff, UK; 3 Bristol Randomised Trials Collaboration, University of Bristol, Bristol, UK; 4 Centre for Exercise, Nutrition and Health Sciences, School for Policy Studies, University of Bristol, Bristol, UK

**Keywords:** Pre-school children, Childcare, Nutrition, Physical activity, Surveys and questionnaires

## Abstract

**Objective:**

To determine the acceptability, internal consistency and test–retest reliability of self-efficacy, motivation and knowledge scales relating to pre-school children’s nutrition, oral health and physical activity.

**Design:**

An online questionnaire was completed twice with an interval of 7–11d.

**Setting:**

Online questionnaires were sent to participants via email from nursery managers. The parent questionnaire was also available on the parenting website www.netmums.com.

**Participants:**

Eighty-two parents and sixty-nine nursery staff from Bristol, UK who had and worked with 2–4-year-olds, respectively.

**Results:**

Response rates were 86·3 and 86·0 % and missing data 15·9 and 14·5 % for the second administration of the parent and nursery staff questionnaires, respectively. Weighted *κ* coefficients for individual items mostly fell under the ‘moderate’ agreement category for the parental (75·0 %) and nursery staff (55·8 %) items. All self-efficacy and motivation scales had acceptable levels of internal consistency (Cronbach’s *α* coefficients>0·7). The intraclass correlation coefficients for the self-efficacy, motivation and knowledge scales ranged between 0·48 and 0·82. Paired *t* tests found an increase between test and retest knowledge scores for the Nutrition Motivation (*t*=−2·91, df=81, *P*=0·00) and Knowledge (*t*=−3·22, df=81, *P*=0·00) scales in the parent questionnaire.

**Conclusions:**

Our findings demonstrate that the items and scales show good acceptability, internal consistency and test–retest reliability.

Globally, an estimated 38·3 million (5·6 %) children under 5 years of age were overweight in 2017^(^ ^1^ ^)^. Guidance and support for caregivers and childcare settings to provide healthy diets and physical activity opportunities have been identified as strategies to reduce the prevalence of obesity in children of pre-school age^(^ ^2^ ^)^. Parents of pre-school children can make certain foods available and accessible in the home environment to promote positive food behaviours^(^ ^3^ ^–^ ^5^ ^)^ and parental encouragement and beliefs about physical activity are important predictors of children’s physical activity levels^(^ ^6^ ^,^ ^7^ ^)^. Various studies have reported that childcare policies have influenced children’s dietary intake and that pre-schools have a responsibility to assist parents in providing healthy food to children^(^ ^8^ ^)^. Childcare staff can also influence the level of physical activity children engage in by encouraging them to be active^(^ ^9^ ^)^. Early childhood caries is a global pandemic and the prevalence among children aged 3–5 years varies between different countries and continents^(^ ^10^ ^)^. Parents and pre-school staff need to supervise and be trained in tooth-brushing practices, together with reducing children’s consumption of sugary foods and drinks, to prevent the onset of early childhood caries^(^ ^10^ ^)^. Parental and family dental health habits influence their children’s oral health^(^ ^11^ ^)^. In the UK about 71 % of eligible 2-year-olds and 95 % of 3–4-year-olds receive government-funded early education in the UK^(^ ^12^ ^)^.

As parents’ and nursery (pre-school) staff’s encouragement have been associated with the quality of children’s diet, oral health and level of physical activity, interventions attempt to increase caregivers’ self-efficacy, motivation and knowledge to improve these behaviours^(^ ^11^ ^,^ ^13^ ^)^. Self-efficacy, a strong predictor of health behaviour change^(^ ^14^ ^)^, is defined as confidence in one’s ability to perform the target behaviour and is a construct of Bandura’s Social Cognitive Theory^(^ ^13^ ^,^ ^15^ ^)^. Motivation refers to one’s readiness to change a specific behaviour, which is defined as the degree to which a person feels a change is important^(^ ^16^ ^,^ ^17^ ^)^. Parental and nursery staff’s knowledge of healthy diets and physical activity may also help encourage children to engage in healthy eating and physical activity^(^ ^18^ ^)^. We are not aware of parent and/or nursery staff questionnaires which measure a combination of attitudes and knowledge towards pre-school children’s nutrition and physical activity. Thus the aims of the current study were to test the Nutrition and Physical Activity Self-Assessment for Child Care (NAP SACC) UK mediators for: (i) acceptability, by examining response rates and missing data; (ii) maximising the internal consistency of the scales using Cronbach’s *α* coefficients; and (iii) assessing the levels of test–retest reliability of individual items and scales using weighted *κ* coefficients, intraclass correlation coefficients (ICC) and paired *t* tests.

## Methods

### Sample

Nurseries from Bristol, UK were identified using the www.1bigdatabase.org.uk and recruited through postal invitations followed by an email invitation 10d later. Participating nursery managers (*n* 21) recruited nursery staff and parents via email. Parents were also recruited via an online advert on the survey forum of the UK-based parenting website www.netmums.com. Data were collected between November 2016 and January 2017. Inclusion criteria were nursery staff and parents or guardians who work with or have 2–4-year-old children. Written informed consent was obtained from the nursery managers and online consent was gained from each participant prior to data collection commencing.

### Study design

Nursery managers were instructed to send a link to the online nursery staff questionnaire via email to all nursery staff who worked with 2–4-year-olds. This was repeated for the parent questionnaire to parents who had 2–4-year-old children. Participants were asked to provide their email address at the end of the questionnaire; those who did were automatically sent the questionnaire again a week later. They were sent a reminder email a further 3d later. Participants’ questionnaires were included in the analyses if the second administration was completed between 7 and 11d after the first administration. Each participant was reimbursed with a £10 voucher on completion of the first and second administrations of the questionnaire.

### Development of the mediator questions

The NAP SACC intervention was designed in the USA to improve the nutrition and physical activity environment, policies and practices in nursery settings^(^ ^19^ ^)^. The aim of the NAP SACC UK feasibility cluster-randomised trial was to assess the acceptability of the intervention, randomisation and study measures within the UK^(^ ^20^ ^)^. A set of potential mediator questions was created for the NAP SACC UK study to measure parents’ and nursery staff’s knowledge, motivation and self-efficacy towards children’s physical activity, oral health, nutrition and sedentary behaviours^(^ ^20^ ^)^. The mediator questions (see online supplementary material) were based on the questionnaire items used in the Active for Life Year 5^(^ ^21^ ^)^ study and were adapted using the best practice of diet as recommended by the Children’s Food Trust^(^ ^22^ ^)^ and UK physical activity guidelines^(^ ^23^ ^)^. The self-efficacy, motivation and knowledge items were split into two sections: children’s nutrition/oral health and children’s physical activity. All the self-efficacy items started with the same stem, ‘I feel able to’, and were followed by dietary-, physical activity- or oral health-related behaviours where the response options were: 1=‘disagree a lot’; 2=‘disagree a little’; 3=‘not sure’; 4=‘agree a little’; and 5=‘agree a lot’. The same health-related behaviours were included in the motivation items but used the stem, ‘I am motivated to’. The motivation response options were: 1=‘never’; 2=‘sometimes’; 3=‘I don’t know’; 4=‘most of the time’; and 5=‘always’. Multiple-choice questions were set for the knowledge items and varied in terms of having one or multiple correct response options.

### Data analysis

Descriptive statistics were used to summarise the participant characteristics, response rates and missing data. Using the data from the first administration of the questionnaire, Cronbach’s *α* coefficients were calculated to determine the internal consistency of the four scales: Nutrition Self-Efficacy, Physical Activity Self-Efficacy, Nutrition Motivation and Physical Activity Motivation. Values of at least 0·7 were considered acceptable^(^ ^24^ ^)^. To assess test–retest reliability of the individual items, weighted *κ* coefficients for ordinal variables^(^ ^25^ ^)^ were calculated. To interpret the *κ* coefficient results, the cut-offs detailed by Landis and Koch^(^ ^26^ ^)^ were used: 0·00–0·20=‘slight’, 0·21–0·40=‘fair’, 0·41–0·60=‘moderate’, 0·61–0·80=‘substantial’ and 0·81–1·00=‘almost perfect’ agreement. A score was derived by calculating the total for each of the self-efficacy and motivation scales. For the knowledge items, the percentage of correct answers was derived for each participant. ICC were used to assess the test–retest agreement at scale level for each of the five scales, with an ICC>0·7 considered acceptable^(^ ^27^ ^)^. The sample size required for estimating an ICC of 0·8 with a 95 % CI ± 0·1 for two repeated measures was fifty participants^(^ ^28^ ^)^. Paired *t* tests were calculated on the continuous test and retest total self-efficacy, motivation and knowledge scale scores to determine whether the scores were higher at the test or retest administration. All analyses were carried out in the statistical software package Stata version 15 (2017).

## Results

### Participants

Eighty-two parents and sixty-nine nursery staff completed the first and second questionnaire administrations within 7–11d and were included in the analyses. Participants’ demographic characteristics are shown in Table 1. Most parents (43·9 %) were in the age group 31–35 years, whereas nursery staff were mainly in the 25–30 years age category (31·9 %). The majority of parents (41·5 %) and nursery staff (37·7 %) had a university degree. The Index of Multiple Deprivation scores of the twenty-one recruited nurseries ranged from 3·59 to 53·27.

**Table 1:** Baseline characteristics of parents and nursery staff who completed two administrations of their respective questionnaires within an interval of 7–11d, Bristol, UK, November 2016–January 2017

	*n*	%
Parent characteristics	*n* 82	
Age (years)		
Under 25	3	3·66
25–30	12	14·63
31–35	36	43·90
36–40	25	30·49
41 or over	6	7·32
Highest level of education		
Did not complete secondary school	1	1·22
GCSE or GNVQ level or equivalent	7	8·54
A levels or Advanced GNVQ or equivalent	9	10·98
University degree	34	41·46
Postgraduate degree or higher	31	37·80
Employment status		
Student	6	7·32
Housewife/househusband	12	14·63
Full-time	21	25·61
Part-time	41	50·00
Unemployed	2	2·44
Number of children, mean and sd	1·68	0·73
Number of children		
1	36	43·90
2	39	47·56
3	4	4·88
4	3	3·66
Nursery staff characteristics	*n* 69	
Age (years)		
Under 25	17	24·64
25–30	22	31·88
31–35	11	15·94
36–40	5	7·25
41 or over	14	20·29
Highest level of education		
GCSE or GNVQ level or equivalent	16	23·19
A levels or Advanced GNVQ or equivalent	21	30·43
University degree	26	37·68
Postgraduate degree or higher	6	8·70

GCSE, General Certificate of Secondary Education; GNVQ, General National Vocational Qualification.

### Acceptability and missing data

The number of times that individuals clicked the consent button on the questionnaire link was 130 and 103 for parents and nursery staff, respectively; it was not possible to distinguish whether the same individuals clicked consent multiple times as they would not have provided any identifying information at this stage (email addresses). One hundred and two parents completed the first administration of the questionnaire and eighty-eight (86·3 %) completed it for the second administration. For the nursery staff questionnaire, eighty-six and seventy-four (86·0 %) participants completed the first and second administrations, respectively.

Seventy-three (89·0 %) and sixty-nine (84·1 %) of the parents completed all items in the first and second questionnaire administrations, respectively. The number of nursery staff completing all the items showed an increase from the first (*n* 57, 82·6 %) to the second (*n* 59, 85·5 %) administration. Thirty-eight (71·7 %) and thirty-four (64·2 %) of the fifty-three parental items had no missing data at test and retest administrations, respectively. Fifty-two (80·0 %) of sixty-five nursery staff questionnaire items had no missing data at both test and retest administrations.

### Cronbach’s α coefficients

Tables 2 and 3 show the Cronbach *α* coefficients of each item for the test scale if the item is removed, as well as the *α* of the overall scale. The Nutrition Self-Efficacy scale showed an acceptable level of internal consistency (*α*=0·80) and the Physical Activity Self-Efficacy scale had the weakest internal consistency in the parent questionnaire but still at an acceptable level (*α*=0·73). The removal of item 17 relating to the provision of opportunities to walk to/from nursery would noticeably improve the internal consistency of the scale (*α*=0·81). The Nutrition Motivation scale showed a high level of internal consistency (*α*=0·86) and the Physical Activity Motivation scale demonstrated the highest overall Cronbach’s *α* (0·89). Unlike the equivalent item in the Physical Activity Self-Efficacy scale, the removal of item 37 had less of an increase on the internal consistency (*α*=0·92). The Nutrition Self-Efficacy and the Nutrition Motivation scales in the nursery staff questionnaire both had *α* coefficients of 0·89, which showed high levels of internal consistency. Both the Physical Activity Self-Efficacy and the Physical Activity Motivation scales also demonstrated high levels of internal consistency (*α*=0·91).

**Table 2:** Cronbach’s *α* coefficients for the four scales in their questionnaire among parents who completed two administrations within an interval of 7–11d, Bristol, UK, November 2016–January 2017

	Cronbach’s *α* if item removed
Nutrition Self-Efficacy Scale	
1. I feel able to provide my children with fruit at all main meals	0·77
2. I feel able to provide my children with vegetables at all main meals	0·78
3. I feel able to reduce the amount of processed meat, fish or potato products served to my children at all main meals	0·78
4. I feel able to provide my children with home-cooked meals each week	0·78
5. I feel able to reduce the number of high-sugar or high-fat snacks served to my children each week	0·76
6. I feel able to reduce the amount of sugary breakfast cereals served to my children each week	0·78
7. I feel able to reduce the number of fizzy drinks and cordials served to my children each week	0·78
8. I feel able to increase the amount of water served to my children each week	0·80
9. I feel able to make changes to the portion sizes served to my children each week	0·79
10. I feel able to increase how often my children brush their teeth with fluoride toothpaste	0·78
Cronbach’s *α* for overall scale	0·80
Physical Activity Self-Efficacy Scale	
11. I feel able to provide my children with time for indoor activities and games each week	0·70
12. I feel able to provide my children with space for indoor activities and games each week	0·68
13. I feel able to provide my children with toys/equipment for indoor activities and games each week	0·71
14. I feel able to provide my children with time for outdoor play and games each week	0·67
15. I feel able to provide my children with space for outdoor play and games each week	0·66
16. I feel able to provide my children with toys/equipment for outdoor play and games each week	0·69
17. I feel able to provide my children with opportunities for walking to/from nursery each week	0·81
18. I feel able to provide my children with opportunities for outdoor play regardless of the weather	0·71
19. I feel able to reduce the amount of time the adults in my household spend using screens across the week	0·73
20. I feel able to reduce the amount of time the children in my household spend using screens across the week	0·72
Cronbach’s *α* for overall scale	0·73
Nutrition Motivation Scale	
21. I am motivated to provide my child with fruit at all main meals	0·85
22. I am motivated to provide my child with vegetables at all main meals	0·85
23. I am motivated to reduce the amount of processed meat, fish or potato products served to my child at all main meals	0·84
24. I am motivated to provide my child with home-cooked meals	0·86
25. I am motivated to reduce the number of high-sugar or high-fat snacks served to my child	0·84
26. I am motivated to reduce the amount of sugary breakfast cereals served to my child	0·84
27. I am motivated to reduce the number of fizzy drinks and cordials served to my child	0·85
28. I am motivated to increase the amount of water served to my child	0·85
29. I am motivated to make changes to the portion sizes served to my child	0·87
30. I am motivated to increase how often my child brushes their teeth with fluoride toothpaste	0·85
Cronbach’s *α* for overall scale	0·86
Physical Activity Motivation Scale	
31. I am motivated to provide my child with time for indoor activities and games	0·88
32. I am motivated to provide my child with space for indoor activities and games	0·87
33. I am motivated to provide my child with toys/equipment for indoor activities and games	0·88
34. I am motivated to provide my child with time for outdoor play and games	0·87
35. I am motivated to provide my child with space for outdoor play and games	0·86
36. I am motivated to provide my child with toys/equipment for outdoor play and games	0·87
37. I am motivated to provide my child with opportunities for walking to/from nursery	0·92
38. I am motivated to provide my child with opportunities for outdoor play regardless of the weather	0·87
39. I am motivated to reduce the amount of time the adults in my household spend using screens	0·89
40. I am motivated to reduce the amount of time the children in my household spend using screens	0·88
Cronbach’s *α* for overall scale	0·89

**Table 3:** Cronbach’s *α* coefficients for the four scales in their questionnaire among nursery staff who completed two administrations within an interval of 7–11d, Bristol, UK, November 2016–January 2017

	Cronbach’s *α* if item removed
Nutrition Self-Efficacy Scale	
1. I feel able to serve fruit and vegetables to children at all main meals	0·89
2. I feel able to limit the amount of processed meat, fish or potato products served to children	0·87
3. I feel able to limit the amount of salt used in food for children	0·87
4. I feel able to limit the number of high-sugar or high-fat snacks served to children	0·88
5. I feel able to limit the use of cakes and/or other sweet or high-fat foods to celebrate events	0·88
6. I feel able to make changes to the types of beverage provided to children	0·87
7. I feel able to make changes to how we promote oral health at nursery	0·88
8. I feel able to make changes to how staff role-model healthy eating foods served at meal and snack times	0·87
9. I feel able to make changes to how staff incorporate healthy eating learning into children’s daily activities	0·87
10. I feel able to increase staff access to professional development in child nutrition	0·88
11. I feel able to increase communication with parents about child nutrition	0·88
12. I feel able to make changes to our written policy on child nutrition	0·87
Cronbach’s *α* for overall scale	0·89
Physical Activity Self-Efficacy Scale	
13. I feel able to provide an appropriately-sized indoor space for children’s physical activity and play	0·90
14. I feel able to provide appropriate indoor toys and equipment for children’s physical activity and play	0·90
15. I feel able to increase the amount of time provided for indoor physical activity and play for children	0·90
16. I feel able to increase the amount of adult-led indoor physical activity and play for children	0·90
17. I feel able to provide an appropriately-sized outdoor space for children’s physical activity and play	0·90
18. I feel able to provide appropriate outdoor toys and equipment for children’s physical activity and play	0·90
19. I feel able to increase the amount of time provided for outdoor physical activity and play for children	0·90
20. I feel able to increase the amount of adult-led outdoor physical activity and play for children	0·91
21. I feel able to make changes to the amount of screen time allowed in our nursery per child	0·91
22. I feel able to make changes to how staff role-model good physical activity habits	0·90
23. I feel able to make changes to how staff incorporate physical activity learning into children’s daily activities	0·90
24. I feel able to increase staff access to professional development in children’s physical activity	0·90
25. I feel able to increase communication with parents about children’s physical activity	0·90
26. I feel able to make changes to our written policy on children’s physical activity	0·90
Cronbach’s *α* for overall scale	0·91
Nutrition Motivation Scale	
27. I am motivated to serve fruit and vegetables to children at all main meals	0·90
28. I am motivated to limit the amount of processed meat, fish or potato products served to children	0·89
29. I am motivated to limit the amount of salt used in food for children	0·89
30. I am motivated to limit the number of high-sugar or high-fat snacks served to children	0·88
31. I am motivated to limit the use of cakes and/or other sweet or high fat foods to celebrate events	0·88
32. I am motivated to make changes to the types of beverage provided to children	0·88
33. I am motivated to make changes to how we promote oral health at nursery	0·88
34. I am motivated to make changes to how staff role-model healthy eating foods served at meal and snack times	0·89
35. I am motivated to make changes to how staff incorporate healthy eating learning into children’s daily activities	0·88
36. I am motivated to increase staff access to professional development in child nutrition	0·88
37. I am motivated to increase communication with parents about child nutrition	0·89
38. I am motivated to make changes to our written policy on child nutrition	0·89
Cronbach’s *α* for overall scale	0·89
Physical Activity Motivation Scale	
39. I am motivated to provide an appropriately-sized indoor space for children’s physical activity and play	0·90
40. I am motivated to provide appropriate indoor toys and equipment for children’s physical activity and play	0·90
41. I am motivated to increase the amount of time provided for indoor physical activity and play for children	0·90
42. I am motivated to increase the amount of adult-led indoor physical activity and play for children	0·90
43. I am motivated to provide an appropriately-sized outdoor space for children’s physical activity and play	0·90
44. I am motivated to provide appropriate outdoor toys and equipment for children’s physical activity and play	0·90
45. I am motivated to increase the amount of time provided for outdoor physical activity and play for children	0·89
46. I am motivated to increase the amount of adult-led outdoor physical activity and play for children	0·90
47. I am motivated to make changes to the amount of screen time allowed in our nursery per child	0·90
48. I am motivated to make changes to how staff role-model good physical activity habits	0·90
49. I am motivated to make changes to how staff incorporate physical activity learning into children’s daily activities	0·89
50. I am motivated to increase staff access to professional development in children’s physical activity	0·90
51. I am motivated to increase communication with parents about children’s physical activity	0·90
52. I am motivated to make changes to our written policy on children’s physical activity	0·91
Cronbach’s *α* for overall scale	0·91

### Test–retest analyses

Test–retest analyses found that most of the weighted *κ* coefficients for individual items fell under the ‘moderate’ category for the parent (75·0 %) questionnaire and for the nursery staff (55·8 %) questionnaire (Table 4). The parent questionnaire scales demonstrated substantial levels of agreement (ICC=0·62 to 0·80). Overall the nursery staff questionnaire scales demonstrated good levels of test–retest reliability, apart from the Physical Activity Motivation (ICC=0·48) scale which can be in part explained by 50 % of the individual items displaying ‘fair’ test–retest reliability. Paired *t* tests found that self-efficacy, motivation and knowledge scale scores for parents were higher in the questionnaire’s second administration. Paired *t* tests showed strong evidence that the Nutrition Motivation (*t*=−2·91, df=81, *P*=0·00) and Knowledge (*t*=−3·22, df=81, *P*=0·00) scales were substantially higher at the retest administration. Similarly, the nursery staff’s scale scores were all higher in the questionnaire’s retest administration; however, there was no evidence that this increase was substantial.

**Table 4:** Weighted *κ* coefficients of the items, intraclass correlation coefficients (ICC) and paired *t* tests of the test scales among parents and nursery staff who completed two administrations of their respective questionnaires within an interval of 7–11d, Bristol, UK, November 2016–January 2017

		Weighted *κ* coefficients																
		Slight (0–<0·2)		Fair (0·2–<0·4)		Moderate (0·4–<0·6)		Substantial (0·6–<0·8)		Almost perfect (0·8–<1·0)						Paired *t* test		
	No. of items	*n*	%	*n*	%	*n*	%	*n*	%	*n*	%	ICC	95 % CI	Mean difference	95 % CI	*t*	df	*P*
Parent questionnaire scales																		
Nutrition Self-Efficacy	10	0		1	10·0	7	70·0	2	20·0	0		0·80	0·71, 0·87	−0·59	−1·33, 0·16	−1·56	81	0·12
Physical Activity Self-Efficacy	10	0		0		8	80·0	1	10·0	1	10·0	0·76	0·65, 0·84	−0·10	−0·97, 0·77	−0·22	81	0·82
Nutrition Motivation	10	0		2	20·0	7	70·0	1	10·0	0		0·62	0·47, 0·74	−1·74	−2·94, −0·55	−2·91	81	0·00
Physical Activity Motivation	10	0		0		8	80·0	2	20·0	0		0·77	0·66, 0·84	−0·59	−1·63, 0·46	−1·11	81	0·27
Knowledge	13	N/A		N/A		N/A		N/A		N/A		0·74	0·63, 0·82	−2·55	−4·12, −0·97	−3·22	81	0·00
Nursery staff questionnaire scales																		
Nutrition Self-Efficacy	12	0		3	25·0	7	58·3	2	16·7	0		0·82	0·72, 0·88	−1·30	−2·63, 0·02	−1·97	68	0·05
Physical Activity Self-Efficacy	14	0		2	14·3	10	71·4	2	14·3	0		0·78	0·67, 0·86	−0·59	−2·13, 0·94	−0·77	68	0·44
Nutrition Motivation	12	1	8·33	5	41·7	6	50·0	0		0		0·61	0·43, 0·74	−1·25	−2·76, 0·27	−1·64	68	0·10
Physical Activity Motivation	14	0		7	50·0	6	42·8	1	7·14	0		0·48	0·28, 0·65	−1·03	−2·73, 0·68	−1·20	68	0·23
Knowledge	13	N/A		N/A		N/A		N/A		N/A		0·76	0·64, 0·84	−0·61	−2·44, 1·21	−0·67	68	0·50

N/A, not applicable.

## Discussion

In the present paper we found that our parental and nursery staff questionnaires on nutrition-, oral health- and physical activity-related self-efficacy, motivation and knowledge for pre-school children demonstrated high levels of acceptability, with most participants completing the second administration of the questionnaire. Eighty-four per cent of the parents and 86 % of the nursery staff participants completed all the items. When analysing the missing data further, no items were consistently unanswered by multiple participants or between the test and retest administrations of the questionnaires; this indicates that the items were seen to be appropriate.

The self-efficacy and motivation scales demonstrated acceptable and high levels of internal consistency. Removing the item on providing weekly opportunities to walk to/from nursery from the parent questionnaire would improve the internal consistency of the two physical activity scales. Our findings suggest that this item does not fit well within the Physical Activity Self-Efficacy and Physical Activity Motivation scales and could therefore affect the scores produced for these two scales. We advise removing this item from these scales or to include it as a separate item in the questionnaire.

The individual self-efficacy and motivation items demonstrated good levels of test–retest reliability, where over 50 % of the *κ* coefficients were categorised as ‘moderate’ for the parent and nursery staff questionnaires. A handful of items were found to have ‘fair’ and ‘slight’ agreement, which might suggest that participants do not understand the questions or are guessing the answers^(^ ^29^ ^)^. Total scores for the self-efficacy, motivation and knowledge scales were derived for each participant and test–retest analyses were carried out using paired *t* tests. Among the parent population, there was a substantial difference between the test and retest responses for two of the scales. In terms of the Knowledge scale, no substantial test–retest difference was observed when the exact same items were answered by the nursery staff. Differences in the results between the parents and nursery staff may be the result of differences in participant age and education levels but this is unclear due to our limited sample size.

The test–retest correlations of the self-efficacy, motivation and knowledge scales ranged from 0·48 to 0·82 across both the parental and nursery staff questionnaires. Our findings are comparable with findings from the literature looking at similar topic areas and/or populations. In a study by Wright *et al*.^(^ ^13^ ^)^, the 1-week test–retest reliability of parental self-efficacy scales relating to children’s physical activity and dietary behaviours ranged from 0·80 to 0·88. Cronbach’s *α* coefficients for the four scales ranged from 0·80 to 0·88 in two different participant samples. In a study by Whittaker and Cowley^(^ ^30^ ^)^, the ICC of three parenting self-efficacy scales relating to children aged 1–4 years, including a play scale, ranged from 0·77 to 0·95 and the internal consistency ranged from 0·66 to 0·84. The Cronbach’s *α* coefficients and test–retest reliability of a seven-item effort motivation scale was 0·92 and 0·61 for teachers and 0·89 and 0·69 for parents of pre-school children^(^ ^31^ ^)^. Nutrition knowledge scales demonstrated test–retest reliability coefficients between 0·33 and 0·75 in a study by Vereecken *et al*.^(^ ^29^ ^)^. The Cronbach’s *α* coefficients for four oral health-related knowledge, fatalism and self-efficacy measures ranged from 0·76 to 0·91 when measured in mothers of children aged 1–5 years^(^ ^32^ ^)^.

There are no currently existing questionnaires which measure parents’ and nursery staff’s self-efficacy, motivation and knowledge towards pre-school children’s nutrition, oral health and physical activity. Our analyses have demonstrated that the items and scales in our questionnaires are acceptable, internally consistent and reliable. A limitation in our paper and other similar studies is that the analyses were carried out in a single sample, therefore we cannot assume that our results would be reproduced when repeated using different populations. It is important to acknowledge that we were limited with our sample size and characteristics, which are not representative of the general population, and therefore it is uncertain whether these items would be deemed as acceptable to more diverse populations. In the UK, Level 6 qualifications for early years staff are degree level and include Qualified Teacher Status (QTS), Early Years Professional Status (EYPS), Early Years Teacher Status (EYTS) and other early years-related degree-level qualifications^(^ ^33^ ^)^. In England in 2016, 29 % of nursery staff had a minimum of a Level 6 qualification^(^ ^33^ ^)^ compared with our nursery staff sample where 46·4 % of individuals had a university degree or higher (minimum Level 6 qualification). Although the percentage of our nursery staff sample with a university degree was higher than the English average, we believe that this would be a problem only if internal consistency and test–retest reliability would be different in a group who had a lower level of educational achievement. However, we acknowledge that our nursery staff questionnaire results may not be generalisable to early years staff in other countries which have different requirements for early years staff qualifications. We recognise that our results may not be replicated if using paper-based or face-to-face versions of the questionnaires as opposed to the online versions used in the present study. This is important to consider in low- to middle-income countries where device and Internet access may not be available to administer tablet/web-based forms of the questionnaire. However, there is evidence to suggest that acceptability, internal consistency and test–retest reliability outcomes are comparable between paper-based and device/web-based forms of questionnaire administration^(^ ^34^ ^–^ ^36^ ^)^. Due to the limitations stated above, caution needs to be taken when interpreting the magnitude of the results and deciding whether to remove certain items for use in studies.

## Conclusions

The scales provided here are an acceptable and reliable method of assessing parents’ and nursery staff’s self-efficacy, motivation and knowledge about pre-school children’s diet, oral health and physical activity. The items in the questionnaire show low levels of missing data and good levels of acceptability, internal consistency and test–retest reliability. Overall our findings suggest that the questionnaires would be suitable measures in assessing parent and nursery staff levels of self-efficacy, motivation and knowledge.
