# Supplementary material for: Acceptability, internal consistency and test–retest reliability of scales to assess parental and nursery staff’s self-efficacy, motivation and knowledge in relation to pre-school children’s nutrition, oral health and physical activity
Source: Public Health Nutr. 2019 Feb 14;22(6):967–75. doi: 10.1017/S1368980018004111 (PMC6520240; doi:10.1017/S1368980018004111)
Supplement: Supplementary file 1 [file S1368980018004111sup001.docx]

**
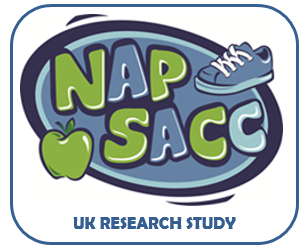
**

**NAP SACC UK QUESTIONNAIRE**

**For Parents**

**Please tick ONE box under the statement that most closely describes how much you agree or disagree with each statement. There are no right or wrong answers, just your opinions about how you feel when caring for your child. These questions are about how much you feel able to do things relating to food and physical activity.**

***Food***

| 1. I feel able to provide my children with fruit at all main meals | *Disagree a lot*  1  2  3  4  5 | *Disagree a little* | *Not sure* | *Agree a little* | *Agree a lot* |
| --- | --- | --- | --- | --- | --- |
| 2. I feel able to provide my children with vegetables at all main meals | *Disagree a lot*  1  2  3  4  5 | *Disagree a little* | *Not sure* | *Agree a little* | *Agree a lot* |
| 3. I feel able to reduce the amount of processed meat, fish or potato products served to my children at all main meals | *Disagree a lot*  1  2  3  4  5 | *Disagree a little* | *Not sure* | *Agree a little* | *Agree a lot* |
| 4. I feel able to provide my children with home-cooked meals each week | *Disagree a lot*  1  2  3  4  5 | *Disagree a little* | *Not sure* | *Agree a little* | *Agree a lot* |
| 5. I feel able to reduce the number of high-sugar or high-fat snacks served to my children each week | *Disagree a lot*  1  2  3  4  5 | *Disagree a little* | *Not sure* | *Agree a little* | *Agree a lot* |
| 6. I feel able to reduce the amount of sugary breakfast cereals served to my children each week | *Disagree a lot*  1  2  3  4  5 | *Disagree a little* | *Not sure* | *Agree a little* | *Agree a lot* |
| 7. I feel able to reduce the number of fizzy drinks and cordials served to my children each week | *Disagree a lot*  1  2  3  4  5 | *Disagree a little* | *Not sure* | *Agree a little* | *Agree a lot* |
| 8. I feel able to increase the amount of water served to my children each week | *Disagree a lot*  1  2  3  4  5 | *Disagree a little* | *Not sure* | *Agree a little* | *Agree a lot* |
| 9. I feel able to make changes to the portion sizes served to my children each week | *Disagree a lot*  1  2  3  4  5 | *Disagree a little* | *Not sure* | *Agree a little* | *Agree a lot* |
| 10. I feel able to increase how often my children brush their teeth with fluoride toothpaste | *Disagree a lot*  1  2  3  4  5 | *Disagree a little* | *Not sure* | *Agree a little* | *Agree a lot* |

***Physical Activity and Play***

| 11. I feel able to provide my children with time for indoor activities and games each week | *Disagree a lot*  1  2  3  4  5 | *Disagree a little* | *Not sure* | *Agree a little* | *Agree a lot* |
| --- | --- | --- | --- | --- | --- |
| 12. I feel able to provide my children with space for indoor activities and games each week | *Disagree a lot*  1  2  3  4  5 | *Disagree a little* | *Not sure* | *Agree a little* | *Agree a lot* |
| 13. I feel able to provide my children with toys/equipment for indoor activities and games each week | *Disagree a lot*  1  2  3  4  5 | *Disagree a little* | *Not sure* | *Agree a little* | *Agree a lot* |
| 14. I feel able to provide my children with time for outdoor play and games each week | *Disagree a lot*  1  2  3  4  5 | *Disagree a little* | *Not sure* | *Agree a little* | *Agree a lot* |
| 15. I feel able to provide my children with space for outdoor play and games each week | *Disagree a lot*  1  2  3  4  5 | *Disagree a little* | *Not sure* | *Agree a little* | *Agree a lot* |
| 16. I feel able to provide my children with toys/equipment for outdoor play and games each week | *Disagree a lot*  1  2  3  4  5 | *Disagree a little* | *Not sure* | *Agree a little* | *Agree a lot* |
| 17. I feel able to provide my children with opportunities for walking to/from nursery each week | *Disagree a lot*  1  2  3  4  5 | *Disagree a little* | *Not sure* | *Agree a little* | *Agree a lot* |
| 18. I feel able to provide my children with opportunities for outdoor play regardless of the weather | *Disagree a lot*  1  2  3  4  5 | *Disagree a little* | *Not sure* | *Agree a little* | *Agree a lot* |
| 19. I feel able to reduce the amount of time the adults in my household spend using screens across the week | *Disagree a lot*  1  2  3  4  5 | *Disagree a little* | *Not sure* | *Agree a little* | *Agree a lot* |
| 20. I feel able to reduce the amount of time the children in my household spend using screens across the week | *Disagree a lot*  1  2  3  4  5 | *Disagree a little* | *Not sure* | *Agree a little* | *Agree a lot* |

**Please tick ONE box under the statement that most closely describes your level of motivation for each statement. There are no right or wrong answers, just your opinions about how you feel when caring for your child.**

***Food***

| 21. I am motivated to provide my child with fruit at all main meals | *Never* | *Sometimes* | *I don’t know* | *Most of the time*  1  2  3  4  5 | *Always* |
| --- | --- | --- | --- | --- | --- |
| 22. I am motivated to provide my child with vegetables at all main meals | *Never* | *Sometimes* | *I don’t know*  1  2  3  4  5 | *Most of the time* | *Always* |
| 23. I am motivated to reduce the amount of processed meat, fish or potato products served to my child at all main meals | *Never* | *Sometimes* | *I don’t know*  1  2  3  4  5 | *Most of the time* | *Always* |
| 24. I am motivated to provide my child with home-cooked meals | *Never* | *Sometimes*  1  2  3  4  5 | *I don’t know* | *Most of the time* | *Always* |
| 25. I am motivated to reduce the number of high-sugar or high-fat snacks served to my child | *Never* | *Sometimes*  1  2  3  4  5 | *I don’t know* | *Most of the time* | *Always* |
| 26. I am motivated to reduce the amount of sugary breakfast cereals served to my child | *Never* | *Sometimes*  1  2  3  4  5 | *I don’t know* | *Most of the time* | *Always* |
| 27. I am motivated to reduce the number of fizzy drinks and cordials served to my child | *Never*  1  2  3  4  5 | *Sometimes* | *I don’t know* | *Most of the time* | *Always* |
| 28. I am motivated to increase the amount of water served to my child | *Never*  1  2  3  4  5 | *Sometimes* | *I don’t know* | *Most of the time* | *Always* |
| 29. I am motivated to make changes to the portion sizes served to my child | *Never* | *Sometimes*  1  2  3  4  5 | *I don’t know* | *Most of the time* | *Always* |
| 30. I am motivated to increase how often my child brushes their teeth with fluoride toothpaste | *Never* | *Sometimes*  1  2  3  4  5 | *I don’t know* | *Most of the time* | *Always* |

***Physical Activity and Play***

| 31. I am motivated to provide my child with time for indoor activities and games | *Never* | *Sometimes*  1  2  3  4  5 | *I don’t know* | *Most of the time* | *Always* |
| --- | --- | --- | --- | --- | --- |
| 32. I am motivated to provide my child with space for indoor activities and games | *Never* | *Sometimes*  1  2  3  4  5 | *I don’t know* | *Most of the time* | *Always* |
| 33. I am motivated to provide my child with toys/equipment for indoor activities and games | *Never* | *Sometimes* | *I don’t know*  1  2  3  4  5 | *Most of the time* | *Always* |
| 34. I am motivated to provide my child with time for outdoor play and games | *Never*  1  2  3  4  5 | *Sometimes* | *I don’t know* | *Most of the time* | *Always* |
| 35. I am motivated to provide my child with space for outdoor play and games | *Never* | *Sometimes*  1  2  3  4  5 | *I don’t know* | *Most of the time* | *Always* |
| 36. I am motivated to provide my child with toys/equipment for outdoor play and games | *Never* | *Sometimes*  1  2  3  4  5 | *I don’t know* | *Most of the time* | *Always* |
| 37. I am motivated to provide my child with opportunities for walking to/from nursery | *Never* | *Sometimes* | *I don’t know*  1  2  3  4  5 | *Most of the time* | *Always* |
| 38. I am motivated to provide my child with opportunities for outdoor play regardless of the weather | *Never* | *Sometimes*  1  2  3  4  5 | *I don’t know* | *Most of the time* | *Always* |
| 39. I am motivated to reduce the amount of time the adults in my household spend using screens | *Never* | *Sometimes*  1  2  3  4  5 | *I don’t know* | *Most of the time* | *Always* |
| 40. I am motivated to reduce the amount of time the children in my household spend using screens | *Never* | *Sometimes*  1  2  3  4  5 | *I don’t know* | *Most of the time* | *Always* |

***These questions are about what you think about children’s food, teeth and physical activity. For each question, please tick all of the options which you agree with:***

**Child food and teeth:**

41. Which of the following food groups should be eaten regularly by 2-4 year old children?

- *Whole grains*
- *Low-fat dairy products*
- *Lean meat and beans*
- *All of the above*

42. How many portions of fruit and vegetables should 2-4 year old children consume per day?

- *3*
- *4*
- *5*
- *More than 5*How

43. What are suitable foods for 2-4 year olds to eat at breakfast?

- Sweetened cereal (e.g. Cheerios, Coco Pops),
- Non-sweetened cereal (e.g. Weetabix, Cornflakes, Porridge),
- Sweetened cereal and toast
- Non-sweetened cereal and toast
- Toast
- Yogurt or fruit
- Milk
- Breakfast is not required

44. What type of puddings should be served to 2-4 year olds?

- Puddings should not be served to children
- Hot fruit-based puddings e.g. crumbles, baked apples
- Milk-based puddings e.g. rice pudding, custard
- Yogurt or fromage frais
- Cakes and biscuits containing fruit e.g. fruit flapjack, carrot cake
- Cold puddings such as fruit salad, piece of fruit
- All of the above

45. What are the recommended drinks for 2-4 year olds?

- Whole milk (full-fat)
- Semi-skimmed milk
- Skimmed milk
- Fruit juice
- Diluted fruit juice
- Water
- Fruit squash/cordial
- Fizzy sweet drinks

46. What are the recommended snacks for 2-4 year olds?

- No snacks between meals
- Dried fruit
- Fresh fruit or vegetables
- Crisps
- Biscuits/cakes
- Breadsticks/sandwich/rice cakes
- Chocolate/sweets

47. How often should 2-4 year old children brush their teeth?

- Twice per day
- Once per day
- After every meal

48. How long should 2-4 year old children brush their teeth each time they brush them?

- 30 seconds
- 1 minute
- 2 minutes

49. At what age is a child able to brush their teeth unsupervised by an adult?

- Age 2
- Age 3
- Age 4
- Age 5
- Age 6
- Age 7
- Age 8

**Child Physical Activity and Play:**

50. How many minutes of active play each day do health professionals recommend for 2-4 year olds?

- 30 minutes
- 45 minutes
- 60 minutes (1 hour)
- 90 minutes
- 120 minutes (2 hours)
- 150 minutes
- 180 minutes (3 hours)

51. When it is raining, children should:

- Stay indoors
- Continue to play outside in whatever they are wearing
- Play outside in wet weather clothes

**Sedentary Time:**

52. How many minutes of screen-viewing each day do health professionals recommend for 2-4 year olds?

o None

o Less than 1 hour

o Between 1-2 hours

o 2-3 hours

o 3-4 hours

o More than 4 hours

53. What are the recommendations for children having TVs in bedrooms

o A TV in a child’s bedroom is ok

o TV in a child’s bedroom helps them to sleep

o Parents should limit the amount of TV watching in a child’s bedroom

o TV in a child’s bedrooms promotes more TV watching

o TVs in a child’s bedrooms makes it more difficult for a child to sleep

o TV in a child’s bedroom can lead to less appropriate viewing

Thank you for completing the questionnaire. Please return it to the NAP SACC UK Study in the stamped addressed envelope to: NAP SACC UK Study (room 4.09), School of Social and Community Medicine, University of Bristol, Canynge Hall, 39 Whatley Road, Bristol BS8 2PS.

**
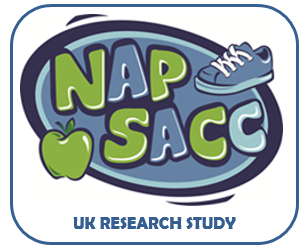
**

**NAP SACC UK QUESTIONNAIRE**

**For Nursery Staff**

**Please tick ONE box under the statement that most closely describes how much you agree or disagree with each statement. There are no right or wrong answers, just your opinions about how you feel when working at your nursery. These questions are about how much you feel able to do things relating to child nutrition and physical activity.**

***Child Nutrition***

| 1. I feel able to serve fruit and vegetables to children at all main meals | *Disagree a lot*  1  2  3  4  5 | *Disagree a little* | *Not sure* | *Agree a little* | *Agree a lot* |
| --- | --- | --- | --- | --- | --- |
| 2. I feel able to limit the amount of processed meat, fish or potato products served to children | *Disagree a lot*  1  2  3  4  5 | *Disagree a little* | *Not sure* | *Agree a little* | *Agree a lot* |
| 3. I feel able to limit the amount of salt used in food for children | *Disagree a lot*  1  2  3  4  5 | *Disagree a little* | *Not sure* | *Agree a little* | *Agree a lot* |
| 4. I feel able to limit the number of high-sugar or high-fat snacks served to children | *Disagree a lot*  1  2  3  4  5 | *Disagree a little* | *Not sure* | *Agree a little* | *Agree a lot* |
| 5. I feel able to limit the use of cakes and/or other sweet or high fat foods to celebrate events | *Disagree a lot*  1  2  3  4  5 | *Disagree a little* | *Not sure* | *Agree a little* | *Agree a lot* |
| 6. I feel able to make changes to the types of beverage provided to children | *Disagree a lot*  1  2  3  4  5 | *Disagree a little* | *Not sure* | *Agree a little* | *Agree a lot* |
| 7. I feel able to make changes to how we promote oral health at nursery | *Disagree a lot*  1  2  3  4  5 | *Disagree a little* | *Not sure* | *Agree a little* | *Agree a lot* |
| 8. I feel able to make changes to how staff role-model healthy eating foods served at meal and snack times | *Disagree a lot*  1  2  3  4  5 | *Disagree a little* | *Not sure* | *Agree a little* | *Agree a lot* |
| 9. I feel able to make changes to how staff incorporate healthy eating learning into children’s daily activities | *Disagree a lot*  1  2  3  4  5 | *Disagree a little* | *Not sure* | *Agree a little* | *Agree a lot* |
| 10. I feel able to increase staff access to professional development in child nutrition | *Disagree a lot*  1  2  3  4  5 | *Disagree a little* | *Not sure* | *Agree a little* | *Agree a lot* |
| 11. I feel able to increase communication with parents about child nutrition | *Disagree a lot*  1  2  3  4  5 | *Disagree a little* | *Not sure* | *Agree a little* | *Agree a lot* |
| 12. I feel able to make changes to our written policy on child nutrition | *Disagree a lot*  1  2  3  4  5 | *Disagree a little* | *Not sure* | *Agree a little* | *Agree a lot* |

***Child Physical Activity and Play***

| 13. I feel able to provide an appropriately-sized indoor space for children’s physical activity and play | *Disagree a lot*  1  2  3  4  5 | *Disagree a little* | *Not sure* | *Agree a little* | *Agree a lot* |
| --- | --- | --- | --- | --- | --- |
| 14. I feel able to provide appropriate indoor toys and equipment for children’s physical activity and play | *Disagree a lot*  1  2  3  4  5 | *Disagree a little* | *Not sure* | *Agree a little* | *Agree a lot* |
| 15. I feel able to increase the amount of time provided for indoor physical activity and play for children | *Disagree a lot*  1  2  3  4  5 | *Disagree a little* | *Not sure* | *Agree a little* | *Agree a lot* |
| 16. I feel able to increase the amount of adult-led indoor physical activity and play for children | *Disagree a lot*  1  2  3  4  5 | *Disagree a little* | *Not sure* | *Agree a little* | *Agree a lot* |
| 17. I feel able to provide an appropriately-sized outdoor space for children’s physical activity and play | *Disagree a lot*  1  2  3  4  5 | *Disagree a little* | *Not sure* | *Agree a little* | *Agree a lot* |
| 18. I feel able to provide appropriate outdoor toys and equipment for children’s physical activity and play | *Disagree a lot*  1  2  3  4  5 | *Disagree a little* | *Not sure* | *Agree a little* | *Agree a lot* |
| 19. I feel able to increase the amount of time provided for outdoor physical activity and play for children | *Disagree a lot*  1  2  3  4  5 | *Disagree a little* | *Not sure* | *Agree a little* | *Agree a lot* |
| 20. I feel able to increase the amount of adult-led outdoor physical activity and play for children | *Disagree a lot*  1  2  3  4  5 | *Disagree a little* | *Not sure* | *Agree a little* | *Agree a lot* |
| 21. I feel able to make changes to the amount of screen-time allowed in our nursery per child | *Disagree a lot*  1  2  3  4  5 | *Disagree a little* | *Not sure* | *Agree a little* | *Agree a lot* |
| 22. I feel able to make changes to how staff role-model good physical activity habits | *Disagree a lot*  1  2  3  4  5 | *Disagree a little* | *Not sure* | *Agree a little* | *Agree a lot* |
| 23. I feel able to make changes to how staff incorporate physical activity learning into children’s daily activities | *Disagree a lot*  1  2  3  4  5 | *Disagree a little* | *Not sure* | *Agree a little* | *Agree a lot* |
| 24. I feel able to increase staff access to professional development in children’s physical activity | *Disagree a lot*  1  2  3  4  5 | *Disagree a little* | *Not sure* | *Agree a little* | *Agree a lot* |
| 25. I feel able to increase communication with parents about children’s physical activity | *Disagree a lot*  1  2  3  4  5 | *Disagree a little* | *Not sure* | *Agree a little* | *Agree a lot* |
| 26. I feel able to make changes to our written policy on children’s physical activity | *Disagree a lot*  1  2  3  4  5 | *Disagree a little* | *Not sure* | *Agree a little* | *Agree a lot* |

**Please tick ONE box under the statement that most closely describes your level of motivation for each statement. There are no right or wrong answers, just your opinions about how you feel when working at your nursery. These questions about your motivation relating to child nutrition and physical activity.**

***Child Nutrition***

| 27. I am motivated to serve fruit and vegetables to children at all main meals | *Never* | *Sometimes*  1  2  3  4  5 | *I don’t know* | *Most of the time* | *Always* |
| --- | --- | --- | --- | --- | --- |
| 28. I am motivated to limit the amount of processed meat, fish or potato products served to children | *Never* | *Sometimes* | *I don’t know* | *Most of the time*  1  2  3  4  5 | *Always* |
| 29. I am motivated to limit the amount of salt used in food for children | *Never* | *Sometimes* | *I don’t know* | *Most of the time*  1  2  3  4  5 | *Always* |
| 30. I am motivated to limit the number of high-sugar or high-fat snacks served to children | *Never* | *Sometimes* | *I don’t know* | *Most of the time*  1  2  3  4  5 | *Always* |
| 31. I am motivated to limit the use of cakes and/or other sweet or high fat foods to celebrate events | *Never* | *Sometimes* | *I don’t know* | *Most of the time*  1  2  3  4  5 | *Always* |
| 32. I am motivated to make changes to the types of beverage provided to children | *Never* | *Sometimes* | *I don’t know* | *Most of the time*  1  2  3  4  5 | *Always* |
| 33. I am motivated to make changes to how we promote oral health at nursery | *Never* | *Sometimes* | *I don’t know* | *Most of the time*  1  2  3  4  5 | *Always* |
| 34. I am motivated to make changes to how staff role-model healthy eating foods served at meal and snack times | *Never* | *Sometimes* | *I don’t know* | *Most of the time*  1  2  3  4  5 | *Always* |
| 35. I am motivated to make changes to how staff incorporate healthy eating learning into children’s daily activities | *Never* | *Sometimes* | *I don’t know* | *Most of the time*  1  2  3  4  5 | *Always* |
| 36. I am motivated to increase staff access to professional development in child nutrition | *Never* | *Sometimes* | *I don’t know* | *Most of the time* | *Always* |
| 37. I am motivated to increase communication with parents about child nutrition | *Never* | *Sometimes*  1  2  3  4  5 | *I don’t know* | *Most of the time* | *Always* |
| 38. I am motivated to make changes to our written policy on child nutrition | *Never* | *Sometimes*  1  2  3  4  5 | *I don’t know* | *Most of the time* | *Always* |

1

2

3

4

5

***Child Physical Activity and Play***

| 39. I am motivated to provide an appropriately-sized indoor space for children’s physical activity and play | *Never* | *Sometimes* | *I don’t know* | *Most of the time*  1  2  3  4  5 | *Always* |
| --- | --- | --- | --- | --- | --- |
| 40. I am motivated to provide appropriate indoor toys and equipment for children’s physical activity and play | *Never* | *Sometimes* | *I don’t know* | *Most of the time*  1  2  3  4  5 | *Always* |
| 41. I am motivated to increase the amount of time provided for indoor physical activity and play for children | *Never* | *Sometimes* | *I don’t know* | *Most of the time*  1  2  3  4  5 | *Always* |
| 42. I am motivated to increase the amount of adult-led indoor physical activity and play for children | *Never* | *Sometimes* | *I don’t know* | *Most of the time*  1  2  3  4  5 | *Always* |
| 43. I am motivated to provide an appropriately-sized outdoor space for children’s physical activity and play | *Never* | *Sometimes* | *I don’t know* | *Most of the time*  1  2  3  4  5 | *Always* |
| 44. I am motivated to provide appropriate outdoor toys and equipment for children’s physical activity and play | *Never* | *Sometimes* | *I don’t know* | *Most of the time*  1  2  3  4  5 | *Always* |
| 45. I am motivated to increase the amount of time provided for outdoor physical activity and play for children | *Never* | *Sometimes* | *I don’t know* | *Most of the time*  1  2  3  4  5 | *Always* |
| 46. I am motivated to increase the amount of adult-led outdoor physical activity and play for children | *Never* | *Sometimes* | *I don’t know* | *Most of the time*  1  2  3  4  5 | *Always* |
| 47. I am motivated to make changes to the amount of screen-time allowed in our nursery per child | *Never* | *Sometimes* | *I don’t know* | *Most of the time*  1  2  3  4  5 | *Always* |
| 48. I am motivated to make changes to how staff role-model good physical activity habits | *Never* | *Sometimes* | *I don’t know* | *Most of the time*  1  2  3  4  5 | *Always* |
| 49. I am motivated to make changes to how staff incorporate physical activity learning into children’s daily activities | *Never* | *Sometimes* | *I don’t know* | *Most of the time*  1  2  3  4  5 | *Always* |
| 50. I am motivated to increase staff access to professional development in children’s physical activity | *Never* | *Sometimes* | *I don’t know* | *Most of the time*  1  2  3  4  5 | *Always* |
| 51. I am motivated to increase communication with parents about children’s physical activity | *Never* | *Sometimes* | *I don’t know* | *Most of the time*  1  2  3  4  5 | *Always* |
| 52. I am motivated to make changes to our written policy on children’s physical activity | *Never* | *Sometimes* | *I don’t know* | *Most of the time*  1  2  3  4  5 | *Always* |

***These questions are about what you think about child nutrition, teeth and physical activity. For each question, please tick all of the options which you agree with:***

**Child Nutrition and Teeth:**

53. Which of the following food groups should be eaten regularly by 2-4 year old children?

- *Whole grains*
- *Low-fat dairy products*
- *Lean meat and beans*
- *All of the above*

54. How many portions of fruit and vegetables should 2-4 year old children consume per day?

- *3*
- *4*
- *5*
- *More than 5*How

55. What are suitable foods for 2-4 year olds to eat at breakfast?

- Sweetened cereal (e.g. Cheerios, Coco Pops),
- Non-sweetened cereal (e.g. Weetabix, Cornflakes, Porridge),
- Sweetened cereal and toast
- Non-sweetened cereal and toast
- Toast
- Yogurt or fruit
- Milk
- Breakfast is not required

56. What type of puddings should be served to 2-4 year olds?

- Puddings should not be served to children
- Hot fruit-based puddings e.g. crumbles, baked apples
- Milk-based puddings e.g. rice pudding, custard
- Yogurt or fromage frais
- Cakes and biscuits containing fruit e.g. fruit flapjack, carrot cake
- Cold puddings such as fruit salad, piece of fruit
- All of the above

57. What are the recommended drinks for 2-4 year olds?

- Whole milk (full-fat)
- Semi-skimmed milk
- Skimmed milk
- Fruit juice
- Diluted fruit juice
- Water
- Fruit squash/cordial
- Fizzy sweet drinks

58. What are the recommended snacks for 2-4 year olds?

- No snacks between meals
- Dried fruit
- Fresh fruit or vegetables
- Crisps
- Biscuits/cakes
- Breadsticks/sandwich/rice cakes
- Chocolate/sweets

59. How often should 2-4 year old children brush their teeth?

- Twice per day
- Once per day
- After every meal

60. How long should 2-4 year old children brush their teeth each time they brush them?

- 30 seconds
- 1 minute
- 2 minutes

61. At what age is a child able to brush their teeth unsupervised by an adult?

- Age 2
- Age 3
- Age 4
- Age 5
- Age 6
- Age 7
- Age 8

**Child Physical Activity and Play:**

62. How many minutes of active play each day do health professionals recommend for 2-4 year olds?

- 30 minutes
- 45 minutes
- 60 minutes (1 hour)
- 90 minutes
- 120 minutes (2 hours)
- 150 minutes
- 180 minutes (3 hours)

63. When it is raining, children should:

- Stay indoors
- Continue to play outside in whatever they are wearing
- Play outside in wet weather clothes

**Sedentary Time:**

64. How many minutes of screen-viewing each day do health professionals recommend for 2-4 year olds?

o None

o Less than 1 hour

o Between 1-2 hours

o 2-3 hours

o 3-4 hours

o More than 4 hours

65. What are the recommendations for children having TVs in bedrooms

o A TV in a child’s bedroom is ok

o TV in a child’s bedroom helps them to sleep

o Parents should limit the amount of TV watching in a child’s bedroom

o TV in a child’s bedrooms promotes more TV watching

o TVs in a child’s bedrooms makes it more difficult for a child to sleep

o TV in a child’s bedroom can lead to less appropriate viewing

Thank you for completing the questionnaire. Please return it to the NAP SACC UK Study in the stamped addressed envelope to: NAP SACC UK Study (room 4.09), School of Social and Community Medicine, University of Bristol, Canynge Hall, 39 Whatley Road, Bristol BS8 2PS.
